# Supplementary material for: Intention to comply with solid waste management practices among households in Butajira town, Southern Ethiopia using the theory of planned behavior
Source: PLoS One. 2022 Jul 8;17(7):e0268674. doi: 10.1371/journal.pone.0268674 (PMC9269971; doi:10.1371/journal.pone.0268674)
Supplement: S1 File — (DOCX) [file pone.0268674.s001.docx]

Annex I: Amharic version of participant information sheet and consent form for TPB phase

(በጥናቱ ለሚሳተፉተ ሳታፊዎች የተዘጋጀ የመረጃ ቅጽ እና የስምምነት ሰነድ)

ስሜ----------------አባላለሁ፡፡ እኔ እዚህ የተገኘሁት በዋቸሞ ዩኒቨርሲቲ በህብረተሰብ ጤና ሁለተኛ ድግሪውን (MPH) እየሰራ ላለው አቶ ሰሙ ደበበ ፍቃዱ ለሚያደረገው ጥናታዊ ፅሁፍ መረጃ አሰባሰብን ለማመቻቸት ነው፡፡

ይህ ቅፅ “በቡታጅራ ከተማ ውስጥ የሚገኙ አባወራዎች ወይም እማወራዎች በቤት ውስጥ የደረቅ ቆሻሻ አያያዝ እና አስተዳደር ላይ ያላቸዉን የወደፊት አላማ (future intention) በሚል ርዕስ ለሚዘጋጀዉ የመመረቂያ ፅሁፍ የቃለ መጠይቅ ተደራጊዎች የፍቃደኝነት መግለጫ ቅፅ ነው፡፡

የጥናቱ ዓላማ እና ጥቅሞቹ ፡- የዚህ ጥናት ዓላማ የአባወራዎች ወይም እማወራዎች በቤት ውስጥ የደረቅ ቆሻሻ አያያዝ እና አስተዳደር ላይ ያላቸዉን የወደፊት አላማ (future intention) መገምገም ነው ፡፡ ከጥናቱ የሚገኘዉ ውጤት አባወራዎች ወይም እማወራዎች የቤት ውስጥ ደረቅ ቆሻሻ አያያዝ እና አስተዳደር ላይ ያላቸዉን ንቃተ-ህሊና ለማሳደግ ሲባል የሚቀረጹ ስትራቴጂዎች የመረጃ ግብዓት ሆኖ ሊያገለግል ይችላል ፡፡ ከዚህም በተጨማሪ ጥናቱ የሚካሄደዉ ለአቶ ሰሙ ደበበ ፍቃዱ የድህረ-ምረቃ ትምህርቱን ጨርሶ ለመመረቅ አስፈላጊ የሆነዉን የመመረቂያ ፅሁፍ ለማዘጋጀት ነው ፡፡

በፍቃደኝነት ላይ የተመሰረተ ተሳትፎ ማቓረጥ ፍራቻ እና አለመመቸት ፡-

ቃለ መጠይቁ ከ20-30 ደቂቃ ይወስዳል ፡፡ ማንኛዉም ተሳትፎ በፈቃደኝነት ላይ የተመሰረተ ሲሆን በማንኛውም ጊዜ ማቓረጥ ይቻላል ፡፡ ይህን ቃለ መጠይቅ ስትጠየቁ ከጊዜያችሁ በቀር የምታወጡት ሌላ ምንም አይነት ወጪ የለም ፡፡ የምታገኙት የገንዘብም ሆነ የተለየ ግላዊ ጥቅም የለም ፡፡ ለመሳተፍ ፍላጎት ካላሳያችሁም የምትጠየቁበት አይሆንም ፡፡ ሆኖም ብትሳተፉ ብዙ ነገር ልታበረክቱልን እንደምትችሉ አውቃችሁ በጥናቱ እንደምትሳተፉልኝ ከልቤ ተስፋ አደርጋለሁ ፡፡ በጥናቱ ለመሳተፍ ከወሰናችሁ እና ከጥያቄዎቹ መካከል መመለስ የማትፈልጉት ጥያቄ ከተጠየቃችሁ ሳትመልሱት መዝለል ወይም ማቆም ትችላላችሁ ፡፡

ምስጥራዊነት፡- ስማችሁ እና ማንነታችሁ ለጥናቱ አያስፈልገንም ፡፡ በመሆኑም ከናንተ የምንሰበስበው ማንኛውም መረጃ በፍፁም ምስጢር የሚያዝ ሲሆን በዚህ ጥናት ላይ ከሚሣተፉ ሠዎች በስተቀር ለሌላ ሦስተኛ ወገን በጭራሽ የማይተላለፍ ነው ፡፡ ለትብብራችሁ በጣም እያመሰገንኩ ጥያቄዎች ካላችሁ በደስታ ለማብራራት ዝግጁ ነን ፡፡ ለተጨማሪ መረጃ ከዚህ ቀጥሎ ያሉትን አድራሻዎች ይጠቀሙ ፡- ስልክ፡- +251912749674 ኢ-ሜይል፡- [semudebe@gmail.com](mailto:semudebe@gmail.com)

ወደ መጠይቁ መቀጠል እንድንችል ፍቃደኛ ነዎት ? 1. አዎ፤ 2. አይደለም

የተሳትፎ ስምምነት፡-ከዚህ በላይ የተዘረዘሩትን መረጃዎች በሙሉ አንብቢያቸዋለሁ ወይም ተነበዉልኛል ፡፡ ጥያቄዎች ለመጠየቅ ዕድል የነበረኝ ሲሆን የጠየቅኳቸዉ ጥያቄዎች በአጥጋቢ ሁኔታ ተመልሰውልኛል ፡፡ ተሳትፎዬን በማንኛዉም ጊዜ የማቓረጥ መብቴ እንደ ተጠበቀ ሆኖ በዚህ ጥናት ቃለ መጠይቅ ላይ ለመሳተፍ በገዛ ፍቃዴ የመረጥኩ መሆኔን አረጋግጣለሁ፡፡

የቃለ መጠይቅ ተደራጊ ፊርማ ፡----------------------------- ቀን፡------------------------------

የመረጃ ሰብሳቢ ፊርማ ፡------------------------------------- ቀን፡------------------------------

**Annex J: Questionnaire Form: Amharic Version for TPB phase**

የቤተሰብ መለያ ቁጥር -----------------

| ጥ.ቁ | | | | ጥያቄዎች | መልስ | | | | | | | |  |  |
| --- | --- | --- | --- | --- | --- | --- | --- | --- | --- | --- | --- | --- | --- | --- |
| **Part 1 . Socio demographic questionnaire** | | | | | | | | | | | | |  |  |
| a. | | | እድሜ ስንት ነው? (በአመት ) | | 1.18 – 25 ዓመት 2. 26 - 35 ዓመት 3. 36 – 45 ዓመት 4. ከ 45 ዓመት በላይ | | | | | | | |  |  |
| b. | | | ፆታ ? | | 1. ወንድ 2. ሴት | | | | | | | |  |  |
| c. | | | ብሄር ? | | 1. ጉራጌ 2. አማራ 3. ስልጤ 4. ኦሮሞ 5. ሌላ | | | | | | | |  |  |
| d. | | | የቤተሰብ አባላት ብዛት ? | | 1. 1-3 ቤተሰብ 2. 4-6 ቤተሰብ 3. 7-8 ቤተሰብ 4. ከ 8 በላይ | | | | | | | |  |  |
| e. | | | ሀይማኖት ? | | 1.ኦርቶዶክስ 2. ሙስሊም 3. ፕሮቴስታንት 4. ካቶሊክ 5. ሌላ____ | | | | | | | |  |  |
| f. | | | የትምህርት ደረጃ ? | | 1.ማንበብ እና መጻፍ የማይችል 2. አንደኛ ደረጃ (1ኛ -8ኛክፍል) 3. ሁለተኛ ደረጃት/ቤት(9ኛ -10ኛ ክፍል ) 4. ፕሪፓራቶሪት/ቤት (11ኛ -12ኛክፍል) 5. ዲፕሎማ 6. የመጀመሪያ ድግሪ 7. 2ኛ ድግሪ | | | | | | | |  |  |
| g. | | | የጋብቻ ሁኔታ ? | | 1.ያገባ/ች 2. ያሊገባ/ች 3. ያሊገባ/ች ግን አብሮ የሚኖሩ 4. የተፋታ/ች | | | | | | | |  |  |
| h. | | | የስራ አይነት ? | | 1.የቀን ሰራተኛ 2. ገበሬ 3. ነጋዴ 4. ተማሪ 5. የመንግስት ሰራተኛ 6. ሌላ ከሆነ (ይገለፅ)------ | | | | | | | |  |  |
| **Part 2 . The Theory of Planned Behaviour Questionnaire** | | | | | | | | | | | | |  |  |
| **A** | **Direct attitude measurement:-** | | | | | | | | | | | | |  |
| የቤት ውስጥ ደረቅ ቆሻሻ አያያዝና እና አስተዳደር ትግበራን ለሚቀጥለው አንድ አመት በአግባቡ ቢከናውን ለእርሶ የሚሰጦትን ፋይዳ ይግለፁ፡፡  መጥፎ -3 -2 -1 0 +1 +2 +3 ጥሩ  የማያገለግል -3 -2 -1 0 +1 +2 +3 የሚያገለግል  ጎጂ -3 -2 -1 0 +1 +2 +3 ጠቃሚ  የማያስደሰት -3 -2 -1 0 +1 +2 +3 የሚያስደስት | | | | | | | | | | | | | | |
| **B.** | | **Behavioral beliefs measurement (Indirect attitude)** | | | | | | | | | | | | |
| 5. | | የቤት ውስጥ ደረቅ ቆሻሻ አያያዝና እና አስተዳደር ትግበራን እርሶ ለሚቀጥለው አንድ አመት በአግባቡ ቢያከናውኑ ጤነኛ የሆነ የህይወት ኑሮ እንዲኖሮት ይረዳዎታል:: | | | | አልስማማም-3 -2 -1 0 +1 +2 +3 እስማማለሁ | | | | | | | | |
| 6 | | የቤት ውስጥ ደረቅ ቆሻሻ አያያዝና እና አስተዳደር ትግበራን እርሶ ለሚቀጥለው አንድ አመት በአግባቡ ቢያከናውኑ ደረቅ ቆሻሻ ካለማስወገድ ተከትሎ ሊከሰት የሚችል ሞትን ይከላከልሎታል :: | | | | አልስማማም-3 -2 -1 0 +1 +2 +3 እስማማለሁ | | | | | | | | |
| 7 | | የቤት ውስጥ ደረቅ ቆሻሻ አያያዝና እና አስተዳደር ትግበራን እርሶ ለሚቀጥለው አንድ አመት በአግባቡ ቢያከናውኑ በበሽታ የመጠቃት እድል ይቀንስሎታል :: | | | | አልስማማም-3 -2 -1 0 +1 +2 +3 እስማማለሁ | | | | | | | | |
| 8 | | የቤት ውስጥ ደረቅ ቆሻሻ አያያዝና እና አስተዳደር ትግበራን እርሶ ለሚቀጥለው አንድ አመት በአግባቡ ቢያከናውኑ የእርሶ አካባቢ ዋንኛው የበሽታ መከሰቻ ቦታ ይሆናል :: | | | | አልስማማም-3 -2 -1 0 +1 +2 +3 እስማማለሁ | | | | | | | | |
| 9 | | የቤት ውስጥ ደረቅ ቆሻሻ አያያዝና እና አስተዳደር ትግበራን እርሶ ለሚቀጥለው አንድ አመት በአግባቡ ቢያከናውኑየከተማውን ጥራት እንዲጨምር ይረዳል :: | | | | አልስማማም-3 -2 -1 0 +1 +2 +3 እስማማለሁ | | | | | | | | |
| 10 | | “የቤት ውስጥ ደረቅ ቆሻሻ አያያዝና እና አስተዳደር ትግበራን እርሶ ለሚቀጥለው አንድ አመት በአግባቡ ቢያከናውኑ”የቤት ውስጥ ደረቅ ቆሻሻ አያያዝና እና አስተዳደር ትግበራን እርሶ ለሚቀጥለው አንድ አመት በአግባቡ ቢያከናውኑ ጭንቀትን እንዲያስወግዱ እና ለመዝናናት ምቹ ሁኔታ ይፈጥሮለታል:: | | | | አልስማማም-3 -2 -1 0 +1 +2 +3 እስማማለሁ | | | | | | | | |
| 11 | | የቤት ውስጥ ደረቅ ቆሻሻ አያያዝና እና አስተዳደር ትግበራን እርሶ ለሚቀጥለው አንድ አመት በአግባቡ ቢያከናውኑ በማህበረሰቡ እርካት እንዲፈጠር ያደርጋል :: | | | | አልስማማም-3 -2 -1 0 +1 +2 +3 እስማማለሁ | | | | | | | | |
| 12 | | የቤት ውስጥ ደረቅ ቆሻሻ አያያዝና እና አስተዳደር ትግበራን እርሶ ለሚቀጥለው አንድ አመት በአግባቡ ቢያከናውኑ የእርሶን የስራ ጊዜ ይሻማዎታል:: | | | | አልስማማም-3 -2 -1 0 +1 +2 +3 እስማማለሁ | | | | | | | | |
| **C** | | **Evaluation of outcomes ( Indirect attitude)** | | | | | | | | | | | | |
| 13 | | ጤነኛ የሆነ የህይወት ኑሮ ማግኘት ለእርሶ የሚያስገኘው ውጤት ፡- | | | | | | | | መጥፎ -3 -2 -1 0 +1 +2 +3 ጥሩ | | | | |
| 14 | | ደረቅ ቆሻሻ ካለማስወገድ ተከትሎ ሊከሰት የሚችል ሞትን መከላከል ለእርሶ የሚያስገኘው ውጤት ፡- | | | | | | | | መጥፎ -3 -2 -1 0 +1 +2 +3 ጥሩ | | | | |
| 15 | | በበሽታ የመጠቃት እድል መቀነስ ለእርሶ የሚያስገኘው ውጤት ፡- | | | | | | | | መጥፎ -3 -2 -1 0 +1 +2 +3 ጥሩ | | | | |
| 16 | | ዋንኛው የበሽታ ምንጭ መሆን ለእርሶ የሚያስገኘው ውጤት ፡- | | | | | | | | መጥፎ -3 -2 -1 0 +1 +2 +3 ጥሩ | | | | |
| 17 | | የከተማውን ጥራት መጨመር ለእርሶ የሚያስገኘው ውጤት ፡- | | | | | | | | መጥፎ -3 -2 -1 0 +1 +2 +3 ጥሩ | | | | |
| 18 | | የጭንቀት መወገድና እና በምቹ ሁኔታ መዝናናት ለእርሶ የሚያስገኘው ውጤት ፡- | | | | | | | | መጥፎ -3 -2 -1 0 +1 +2 +3 ጥሩ | | | | |
| 19 | | የማህበረሰብ እርካት መፍጠር ለእርሶ የሚያስገኘው ውጤት ፡- | | | | | | | | መጥፎ -3 -2 -1 0 +1 +2 +3 ጥሩ | | | | |
| 20 | | የስራ ጊዜ መሻማት ለእርሶ የሚያስገኘው ውጤት ፡- | | | | | | | | መጥፎ -3 -2 -1 0 +1 +2 +3 ጥሩ | | | | |
| **D.** | | **Direct subjective norm measurement** | | | | | | | | | | | | |
| 21 | | የቤት ውስጥ ደረቅ ቆሻሻ አያያዝና እና አስተዳደር ትግበራን እርሶ ለሚቀጥለው አንድ አመት በአግባቡ እንዲያከናውኑ አብዛኛው ለእርሶ የቅርብ ግንኙነት ያላቸው ሰዎች ተግባሩ እንዲሳካ የአሳብ ያደርጋሉ፡፡ | | | | | | | አልስማማም-3 -2 -1 0 +1 +2 +3 እስማማለሁ | | | | | |
| 22 | | የቤት ውስጥ ደረቅ ቆሻሻ አያያዝና እና አስተዳደር ትግበራን እርሶ ለሚቀጥለው አንድ አመት በአግባቡ ማከናወን ይህ በእርሶ የሚጠበቅ ተግባር ነው፡፡ | | | | | | | አልስማማም-3 -2 -1 0 +1 +2 +3 እስማማለሁ | | | | | |
| 23 | | የቤት ውስጥ ደረቅ ቆሻሻ አያያዝና እና አስተዳደር ትግበራን እርሶ ለሚቀጥለው አንድ አመት በአግባቡ ማከናወኖን በተመለከተ ከእርሶ አካባቢ የሚኖሩ ሰዎች ያላቸው አመለካከት እርሶ የሚሰጡት ዋጋ ይግለፁ ፡፡ | | | | | | | አልስማማም-3 -2 -1 0 +1 +2 +3 እስማማለሁ | | | | | |
| **E.** | | **Normative beliefs measurement ( Indirect subjective norm )** | | | | | | | | | | | | |
| 24 | | የቤት ውስጥ ደረቅ ቆሻሻ አያያዝና እና አስተዳደር ትግበራን እርሶ ለሚቀጥለው አንድ አመት በአግባቡ ቢያከናውኑ የእርሶ ቤተሰቦች ደስተኛ ይሆናሉ፡፡ | | | | | አልስማማም-3 -2 -1 0 +1 +2 +3 እስማማለሁ | | | | | | | |
| 25 | | የቤት ውስጥ ደረቅ ቆሻሻ አያያዝና እና አስተዳደር ትግበራን እርሶ ለሚቀጥለው አንድ አመት በአግባቡ ቢያከናውኑ የእርሶ ጎረቤቶች በተግባሩ ይስማማሉ፡፡ | | | | | አልስማማም-3 -2 -1 0 +1 +2 +3 እስማማለሁ | | | | | | | |
| 26 | | የቤት ውስጥ ደረቅ ቆሻሻ አያያዝና እና አስተዳደር ትግበራን እርሶ ለሚቀጥለው አንድ አመት በአግባቡ ቢያከናውኑ በእርሶ አካባቢ የሚገኙ በፈቃደኝነት የነፃ አገልግሎት ከሚሰጡ ሰዎች ስራው እንዲሳካ ግፊት ይደረግሎታል፡፡ | | | | | አልስማማም-3 -2 -1 0 +1 +2 +3 እስማማለሁ | | | | | | | |
| 27 | | የቤት ውስጥ ደረቅ ቆሻሻ አያያዝና እና አስተዳደር ትግበራን እርሶ ለሚቀጥለው አንድ አመት በአግባቡ ቢያከናውኑ በእርሶ አካባቢ ከሚገኙ ልማዳዊ አመለካከት ያላቸው ፖለቲከኛች በተግባሩ እርካታ አያገኙም ፡፡ | | | | | አልስማማም-3 -2 -1 0 +1 +2 +3 እስማማለሁ | | | | | | | |
| 28 | | የቤት ውስጥ ደረቅ ቆሻሻ አያያዝና እና አስተዳደር ትግበራን እርሶ ለሚቀጥለው አንድ አመት በአግባቡ ቢያከናውኑ በእርሶ አካባቢ ከሚገኙ የጤና ባለሙያዎች ስራው እንዲሳካ ግፊት ይደረግሎታል፡፡ | | | | | አልስማማም-3 -2 -1 0 +1 +2 +3 እስማማለሁ | | | | | | | |
| **F.** | | **Motivation to comply ( Indirect subjective norm )** | | | | | | | | | | | | |
| 29 | | የቤት ውስጥ ደረቅ ቆሻሻ አያያዝና እና አስተዳደር ትግበራን እርሶ ለሚቀጥለው አንድ አመት በአግባቡ ሲያከናውኑ በዚህ ተግባር እርሶ ጠንካራ ስራ እንደሚሰሩ፤ በቤተሰቦ ግምት ውስጥ አለ ፡፡ | | | | | | ያልታሰበ -3 -2 -1 0 +1 +2 +3 የታሰበ | | | | | | |
| 30 | | የቤት ውስጥ ደረቅ ቆሻሻ አያያዝና እና አስተዳደር ትግበራን እርሶ ለሚቀጥለው አንድ አመት በአግባቡ ማከናወን ተግባሩ ለእርሶ ጠቃሚ መሆኑ ፤ በእርሶ ጎረቤቶች ግምት ውስጥ አለ ፡፡ | | | | | | ያልታሰበ -3 -2 -1 0 +1 +2 +3 የታሰበ | | | | | | |
| 31 | | የቤት ውስጥ ደረቅ ቆሻሻ አያያዝና እና አስተዳደር ትግበራን እርሶ ለሚቀጥለው አንድ አመት በአግባቡ እንዲያከናውኑ በፈቃደኛ የነፃ አገልግሎት ከሚሰጡ ሰዎች የሚሰጦት ድጋፍ ፤ ተግባሩ ለእርሶ ጠቃሚ መሆኑ በመገንዘብ ነው ፡፡ | | | | | | ያልታሰበ -3 -2 -1 0 +1 +2 +3 የታሰበ | | | | | | |
| 32 | | የቤት ውስጥ ደረቅ ቆሻሻ አያያዝና እና አስተዳደር ትግበራን እርሶ ለሚቀጥለው አንድ አመት በአግባቡ ሲያከናውኑ በእርሶ አካባቢ ከሚገኙ ልማዳዊ አመለካከት ባላቸው ፖለቲከኛች በጥሩ እይታ ያለመታየት በተግባሩ ላይ ለውጥ ያመጣዎበታል ፡፡ | | | | | | ያልታሰበ -3 -2 -1 0 +1 +2 +3 የታሰበ | | | | | | |
| 33 | | የቤት ውስጥ ደረቅ ቆሻሻ አያያዝና እና አስተዳደር ትግበራን እርሶ ለሚቀጥለው አንድ አመት በአግባቡ እንዲያከናውኑ በጤና ባለሙያዎች የሚደረግሎት ድጋፍ ፤ ተግባሩ ለእርሶ ጠቃሚ መሆኑ በመገንዘብ ነው ፡፡ | | | | | | ያልታሰበ -3 -2 -1 0 +1 +2 +3 የታሰበ | | | | | | |
| **G.** | | **Direct perceived behavioral control measurement** | | | | | | | | | | | | |
| 34 | | የቤት ውስጥ ደረቅ ቆሻሻ አያያዝና እና አስተዳደር ትግበራን እርሶ ለሚቀጥለው አንድ አመት በአግባቡ ለማከናወን በፈለጉበት ጊዜ ተግባሩን ለማከናወን ሙሉ በሙሉ በእርሶ አቅም ይተማመናሉ ፡፡ | | | | | | | | | አልስማማም-3 -2 -1 0 +1 +2 +3 እስማማለሁ | | | |
| 35 | | የቤት ውስጥ ደረቅ ቆሻሻ አያያዝና እና አስተዳደር ትግበራን እርሶ ለሚቀጥለው አንድ አመት በአግባቡ ለማከናወን የሚያረጉት ውሳኔ በእርሶ ቁጥጥር ነው ፡፡ | | | | | | | | | አልስማማም-3 -2 -1 0 +1 +2 +3 እስማማለሁ | | | |
| 36 | | የቤት ውስጥ ደረቅ ቆሻሻ አያያዝና እና አስተዳደር ትግበራን እርሶ ለሚቀጥለው አንድ አመት በአግባቡ ለማከናወን እርሶ ያሎትን ችሎታ ላይ ምንም አይነት ጥርጥር የሎትም ፡፡ | | | | | | | | | አልስማማም-3 -2 -1 0 +1 +2 +3 እስማማለሁ | | | |
| 37 | | የቤት ውስጥ ደረቅ ቆሻሻ አያያዝና እና አስተዳደር ትግበራን እርሶ ለሚቀጥለው አንድ አመት በአግባቡ ለማከናወን ሙሉ በሙሉ በእርሶ ቁጥጥር ስር ነው ፡፡ | | | | | | | | | አልስማማም-3 -2 -1 0 +1 +2 +3 እስማማለሁ | | | |
| 38 | | የቤት ውስጥ ደረቅ ቆሻሻ አያያዝና እና አስተዳደር ትግበራን እርሶ ለሚቀጥለው አንድ አመት በአግባቡ ለማከናወን ለእርሶ ቀላል ነገር ነው | | | | | | | | | አልስማማም-3 -2 -1 0 +1 +2 +3 እስማማለሁ | | | |
| 39 | | የቤት ውስጥ ደረቅ ቆሻሻ አያያዝና እና አስተዳደር ትግበራን እርሶ ለሚቀጥለው አንድ አመት በአግባቡ ለማከናወን ወይም ያለማከናወን ውሳኔው ሙሉ በሙሉ በእርሶ ቁጥጥር ነው ፡፡ | | | | | | | | | አልስማማም-3 -2 -1 0 +1 +2 +3 እስማማለሁ | | | |
| **H.** | | **Control beliefs measurement ( Indirect perceived behavioral control)** | | | | | | | | | | | | |
| 40 | | የቤት ውስጥ ደረቅ ቆሻሻ አያያዝና እና አስተዳደር ትግበራን እርሶ ለሚቀጥለው አንድ አመት በአግባቡ ለማከናወን ድጋፍ እና ማበረታቻ ተግባሮ ለማከናወን ምቹ ሁኔታ ይፈጥርሎታል፡፡ | | | | | | አልስማማም-3 -2 -1 0 +1 +2 +3 እስማማለሁ | | | | | | |
| 41 | | የቤት ውስጥ ደረቅ ቆሻሻ አያያዝና እና አስተዳደር ትግበራን እርሶ ለሚቀጥለው አንድ አመት በአግባቡ ለማከናወን በጣም ምቹ የሆነ ቦታ ተግባሮ ለማከናወን ምቹ ሁኔታ ይፈጥርሎታል፡፡ | | | | | | አልስማማም-3 -2 -1 0 +1 +2 +3 እስማማለሁ | | | | | | |
| 42 | | የቤት ውስጥ ደረቅ ቆሻሻ አያያዝና እና አስተዳደር ትግበራን እርሶ ለሚቀጥለው አንድ አመት በአግባቡ ለማከናወን የግንዛቤ ወይም የእውቀት ክፍተት ተግባሮን ለማከናወን ይገታዎታል፡፡ | | | | | | አልስማማም-3 -2 -1 0 +1 +2 +3 እስማማለሁ | | | | | | |
| 43 | | የቤት ውስጥ ደረቅ ቆሻሻ አያያዝና እና አስተዳደር ትግበራን እርሶ ለሚቀጥለው አንድ አመት በአግባቡ ለማከናወን የሚያስፈልጉ የቁሳቁስ እጥረት ተግባሮን ለማከናወን ይገታዎታል፡፡ | | | | | | አልስማማም-3 -2 -1 0 +1 +2 +3 እስማማለሁ | | | | | | |
| 44 | | የቤት ውስጥ ደረቅ ቆሻሻ አያያዝና እና አስተዳደር ትግበራን እርሶ ለሚቀጥለው አንድ አመት በአግባቡ ለማከናወን ተጨማሪ የስራ ጫና ተግባሮን ለማከናወን ይገታዎታል፡፡ | | | | | | አልስማማም-3 -2 -1 0 +1 +2 +3 እስማማለሁ | | | | | | |
| **I** | | **Power of control measurement ( Indirect perceived behavioral control)** | | | | | | | | | | | | |
| 45 | | ድጋፍ እና ማበረታቻ እርሶ የሚተገብሩት የቤት ውስጥ ደረቅ ቆሻሻ አያያዝና እና አስተዳደር ትግበራን ለሚቀጥለው አንድ አመት በአግባቡ ለማከናወን ያለው የአስተዋፅኦ መጠን ይግለፁት፡፡ | | | | | | | | | | አስቸጋሪ -3 -2 -1 0 +1 +2 +3 ቀላል | | |
| 46 | | በጣም ምቹ የሆነ ቦታ እርሶ የሚተገብሩት የቤት ውስጥ ደረቅ ቆሻሻ አያያዝና እና አስተዳደር ትግበራን ለሚቀጥለው አንድ አመት በአግባቡ ለማከናወን ያለው አስተዋፅኦ መጠን ይግለፁት፡፡ | | | | | | | | | | አስቸጋሪ -3 -2 -1 0 +1 +2 +3 ቀላል | | |
| 47 | | የግንዛቤ ወይም የእውቀት ክፍተት እርሶ የሚተገብሩት የቤት ውስጥ ደረቅ ቆሻሻ አያያዝና እና አስተዳደር ትግበራን ለሚቀጥለው አንድ አመት በአግባቡ ለማከናወን ያለው የአስተዋፅኦ መጠን ይግለፁት፡፡ | | | | | | | | | | አስቸጋሪ -3 -2 -1 0 +1 +2 +3 ቀላል | | |
| 48 | | የሚያስፈልጉ የቁሳቁስ እጥረት እርሶ የሚተገብሩት የቤት ውስጥ ደረቅ ቆሻሻ አያያዝና እና አስተዳደር ትግበራን ለሚቀጥለው አንድ አመት በአግባቡ ለማከናወን ያለው የአስተዋፅኦ መጠን ይግለፁት፡፡ | | | | | | | | | | አስቸጋሪ -3 -2 -1 0 +1 +2 +3 ቀላል | | |
| 49 | | ተጨማሪ የስራ ጫና እርሶ የሚተገብሩት የቤት ውስጥ ደረቅ ቆሻሻ አያያዝና እና አስተዳደር ትግበራን ለሚቀጥለው አንድ አመት በአግባቡ ለማከናወን ያለው የአስተዋፅኦ መጠን ይግለፁት፡፡ | | | | | | | | | | አስቸጋሪ -3 -2 -1 0 +1 +2 +3 ቀላል | | |
| **J.** | | **Behavioral Intention** | | | | | | | | | | | | |
| 50 | | የቤት ውስጥ ደረቅ ቆሻሻ አያያዝና እና አስተዳደር ትግበራን እርሶ ለሚቀጥለው አንድ አመት በአግባቡ ለማከናወን ያሎት እቅድዎ ወይም አላማዎትን ይግለፁ ፡፡ | | | | | | | | | አልስማማም-3 -2 -1 0 +1 +2 +3 እስማማለሁ | | | |
| 51 | | የቤት ውስጥ ደረቅ ቆሻሻ አያያዝና እና አስተዳደር ትግበራን እርሶ ለሚቀጥለው አንድ አመት በአግባቡ ለማከናወን ያሎት ቅድመ ዝግጅት ይግለፁ ፡፡ | | | | | | | | | አልስማማም-3 -2 -1 0 +1 +2 +3 እስማማለሁ | | | |
| 52 | | የቤት ውስጥ ደረቅ ቆሻሻ አያያዝና እና አስተዳደር ትግበራን እርሶ ለሚቀጥለው አንድ አመት በአግባቡ ለማከናወን ያሎት ውሳኔ ይግለፁ ፡፡ | | | | | | | | | አልስማማም-3 -2 -1 0 +1 +2 +3 እስማማለሁ | | | |

የአመቻችስም_____________________________ ፊርማ________________ቀን_______/_____/_____

ያረጋገጠው ሱፐርቫይዘር ስም_________________________

ፊርማ_________________ቀን_____/_____/_____

አረጋግጦ ያፀደቀው የጥናት አድራጊውስም ሰሙ ደበበ ፍቃዱ ፊርማ______________ቀን_____/_____/______

ይህን ቃለ መጠይ ቅፎርም ለመጠየቅ ላወጡት ጊዜና ጉልበት በጣም አመሰግናለው
