# Supplementary material for: Intention to comply with solid waste management practices among households in Butajira town, Southern Ethiopia using the theory of planned behavior
Source: PLoS One. 2022 Jul 8;17(7):e0268674. doi: 10.1371/journal.pone.0268674 (PMC9269971; doi:10.1371/journal.pone.0268674)
Supplement: S2 File — (DOCX) [file pone.0268674.s002.docx]

**Annex F: English version Questionnaire Form** **for TPB phase**

Household Code Number : ------------------------------

| **No** | **Question** | | **Answers** | | | | | | |  |
| --- | --- | --- | --- | --- | --- | --- | --- | --- | --- | --- |
| **Part 1. Socio demographic questionnaire** | | | | | | | | | |  |
|  | Age in years | 1.18 – 25 yrs 2. 26 - 35 yrs 3. 36 – 45 yrs 4. above 45yrs | | | | | | | |  |
|  | Sex : | 1.M 2. F | | | | | | | |  |
|  | Ethinicity ? | 1.Gurahge 2. Amehara 3. Silite 4. Oromo 5.others | | | | | | | |  |
|  | The family size of households? | 1.1-3 Members 2. 4-6 Members 3. 7-8 Members 4. above 8 | | | | | | | |  |
|  | What is your Religion? | 1.Orthodox 2. Muslim 3. Protestant 4.Catholic 5.Others______ | | | | | | | |  |
|  | What is your educational status ? | 1.Unable to read and write 2. Primary education (grade 1-8)  3. Secondary education (grade 9-10) 4. Preparatory education (grad 11-12) 5. Dipeloma 6. 1^st^  degree 7. 2^nd^ degree | | | | | | | |  |
|  | What is your marital status? | 1.Married 2.Single 3.Widowed 4. Divorced | | | | | | | |  |
|  | your occupation types is ? | 1.Daily laborer 2. Farmer 3.Merchant 4.Students 5. Gov't employee 6. Others (Specify) | | | | | | | |  |
| **Part 2 . The Theory of Planned Behaviour Questionnaire** | | | | | | | | | |  |
| **A.** | **Direct attitude measurement:-** | | | | | | | | |  |
| For me, implementing a sustainable solid waste management practice in the households, for the next 12 months would be :-   1. Bad -3 -2 -1 0 +1 +2 +3 Good 2. Useless -3 -2 -1 0 +1 +2 +3 Useful 3. Harmful -3 -2 -1 0 +1 +2 +3 Beneficial 4. Unpleasant -3 -2 -1 0 +1 +2 +3 Pleasant | | | | | | | | | |  |
| **B.** | **Behavioral beliefs measurement (Indirect attitude)** | | | | | | | | | |
| 5. | My implementing a sustainable solid waste management practice in the households, for the next 12 months would support to get healther life: | | | | | | | Unlikely -3 -2 -1 0 +1 +2 +3 Likely | | |
| 6. | My implementing a sustainable solid waste management practice in the households, for the next 12 months would assist to prevent my self from death related to poor waste management : | | | | | | | Unlikely -3 -2 -1 0 +1 +2 +3 Likely | | |
| 7. | My implementing a sustainable solid waste management practice in the households, for the next 12 months would make me Prevent the risk of infection. | | | | | | | Unlikely -3 -2 -1 0 +1 +2 +3 Likely | | |
| 8. | My implementing a sustainable solid waste management practice in the households, for the next 12 months will cause potential source of disease for me: | | | | | | | Unlikely -3 -2 -1 0 +1 +2 +3 Likely | | |
| 9. | My implementing a sustainable solid waste management practice in the households, for the next 12 months would help to promote the quality of the urban environment: | | | | | | | Unlikely -3 -2 -1 0 +1 +2 +3 Likely | | |
| 10. | My implementing a sustainable solid waste management practice in the households, for the next 12 months would help to release stress and promote relaxation : | | | | | | | Unlikely -3 -2 -1 0 +1 +2 +3 Likely | | |
| 11. | My implementing a sustainable solid waste management practice in the households, for the next 12 months would help to maintain community satisfaction : | | | | | | | Unlikely -3 -2 -1 0 +1 +2 +3 Likely | | |
| 12. | My implementing a sustainable solid waste management practice in the households, for the next 12 months would occure to consuming of time for me : | | | | | | | Unlikely -3 -2 -1 0 +1 +2 +3 Likely | | |
| **C.** | **Evaluation of outcomes ( Indirect attitude)** | | | | | | | | | |
| 13. | For me, getting healther life is: | | | | | | | Bad -3 -2 -1 0 +1 +2 +3 Good | | |
| 14. | For me, to prevent my self from death related to poor waste management is: | | | | | | | Bad -3 -2 -1 0 +1 +2 +3 Good | | |
| 15. | For me Prevent the risk of infection is: | | | | | | | Bad -3 -2 -1 0 +1 +2 +3 Good | | |
| 16. | For me, causing potential source of disease is : | | | | | | | Bad -3 -2 -1 0 +1 +2 +3 Good | | |
| 17. | For me, to promote the quality of the urban environment is: | | | | | | | Bad -3 -2 -1 0 +1 +2 +3 Good | | |
| 18. | For me, to release stress and promote relaxation :is: | | | | | | | Bad -3 -2 -1 0 +1 +2 +3 Good | | |
| 19. | For me, to maintain community satisfaction is: | | | | | | | Bad -3 -2 -1 0 +1 +2 +3 Good | | |
| 20. | For me, to consuming of time is: | | | | | | | Bad -3 -2 -1 0 +1 +2 +3 Good | | |
| **D.** | **Direct subjective norm measurement** | | | | | | | | | |
| 21. | Most of the people who are important to me think that I -------------implementing a sustainable solid waste management practice in the households, for the next 12 months . | | | | | Should not -3 -2 -1 0 +1 +2 +3 Should | | | | |
| 22. | It is expected of me that I implement a sustainable solid waste management practice in the households, for the next 12 months . | | | | | Unlikely -3 -2 -1 0 +1 +2 +3 Likely | | | | |
| 23. | The people in my life whose opinions I value would_____of my implementing a sustainable solid waste management practice in the households, for the next 12 months . | | | | | Disapprove -3 -2 -1 0 +1 +2 +3 Approve | | | | |
| **E.** | **Normative beliefs measurement ( Indirect subjective norm )** | | | | | | | | | |
| 24. | My family would be Pleased if I implementing a sustainable “solid waste management practice in the households, for the next 12 months | | | | | | | | Disagree -3 -2 -1 0 +1 +2 +3 Agree | |
| 25. | My neighbour’s would ---- of my implementing a sustainable solid waste management practice in the households, for the next 12 months . | | | | | | | | Disagree -3 -2 -1 0 +1 +2 +3 Agree | |
| 26. | I am under pressure from the Community volunteers to implementing a sustainable solid waste management practice in the households, for the next 12 months . | | | | | | | | Disagree -3 -2 -1 0 +1 +2 +3 Agree | |
| 27. | A politicians /conservatives would be disappointed in me if I did implementing a sustainable solid waste management practice in the households, for the next 12 months . | | | | | | | | Disagree -3 -2 -1 0 +1 +2 +3 Agree | |
| 28. | I am under pressure from the health professional to implementing a sustainable solid waste management practice in the households, for the next 12 months . | | | | | | | | Disagree -3 -2 -1 0 +1 +2 +3 Agree | |
| **F.** | **Motivation to comply ( Indirect subjective norm )** | | | | | | | | | |
| 29. | When it comes to implementing a sustainable solid waste management practice in the households, for the next 12 months I am most keen to do what my family expect. | | | Not at all -3 -2 -1 0 +1 +2 +3 Very much | | | | | | |
| 30. | Implementing a sustainable solid waste management practice in the households, for the next 12 months to my only neighbour’s expect me is, important to me. | | | Not at all -3 -2 -1 0 +1 +2 +3 Very much | | | | | | |
| 31. | The Community volunteers approval of my implementing a sustainable solid waste management practice in the households, for the next 12 months is important to me: | | | Not at all -3 -2 -1 0 +1 +2 +3 Very much | | | | | | |
| 32. | Pleasing a politicians/conservatives by always implementing a sustainable solid waste management practice in the households, for the next 12 months matters a lot to me. | | | Not at all -3 -2 -1 0 +1 +2 +3 Very much | | | | | | |
| 33. | The health professional approval of my implementing a sustainable solid waste management practice in the households, for the next 12 months is important to me: | | | Not at all -3 -2 -1 0 +1 +2 +3 Very much | | | | | | |
| **G.** | **Direct perceived behavioral control measurement** | | | | | | | | | |
| 34. | I am confident that I can implement a sustainable solid waste management practice in the households, for the next 12 months if I want to. | | | | Disagree -3 -2 -1 0 +1 +2 +3 Agree | | | | | |
| 35. | The decision to implement a sustainable solid waste management practice in the households, for the next 12 months is beyond my control. | | | | Disagree -3 -2 -1 0 +1 +2 +3 Agree | | | | | |
| 36. | I have no doubt about my ability to go for implement a sustainable “solid waste management practice in the households, for the next 12 months”. | | | | Disagree -3 -2 -1 0 +1 +2 +3 Agree | | | | | |
| 37. | It is completely up to me if I want to implement a sustainable solid waste management practice in the households, for the next 12 months . | | | | Disagree -3 -2 -1 0 +1 +2 +3 Agree | | | | | |
| 38. | For me to implement a sustainable solid waste management practice in the households, for the next 12 months would be easy. | | | | Disagree -3 -2 -1 0 +1 +2 +3 Agree | | | | | |
| 39. | I have complete control in deciding whether or not to implement a sustainable solid waste management practice in the households, for the next 12 months . | | | | No control -3 -2-1 0 +1 +2 +3 Complete control | | | | | |
| **H.** | **Control beliefs measurement ( Indirect perceived behavioral control)** | | | | | | | | | |
| 40. | I think that support and encouragement would enable me to implement a sustainable solid waste management practice in the households, for the next 12 months . | | | | | | Disagree -3 -2 -1 0 +1 +2 +3 Agree | | | |
| 41. | I expect that the more convenient location would enable me to implement a sustainable solid waste management practice in the households, for the next 12 months . | | | | | | Disagree -3 -2 -1 0 +1 +2 +3 Agree | | | |
| 42. | I expect that lack of information (training/education) about how to implement would prevent me from implementing a sustainable solid waste management practice in the households, for the next 12 months . | | | | | | Disagree -3 -2 -1 0 +1 +2 +3 Agree | | | |
| 43. | I think that lack of equipment availability would prevent me to implement a sustainable solid waste management practice in the households, for the next 12 months . | | | | | | Disagree -3 -2 -1 0 +1 +2 +3 Agree | | | |
| 44. | I expect that the issue of raising workload would prevent me from implementing a sustainable solid waste management practice in the households, for the next 12 months . | | | | | | Disagree -3 -2 -1 0 +1 +2 +3 Agree | | | |
| **I.** | **Power of control measurement ( Indirect perceived behavioral control)** | | | | | | | | | |
| 45. | Support and encouragement would make it------------- for me to to implement a sustainable solid waste management practice in the households, for the next 12 months . | | | Difficult -3 -2 -1 0 +1 +2 +3 easier | | | | | | |
| 46. | More "convenient" location would make it------------- for me to to implement a sustainable “solid waste management practice in the households, for the next 12 months”. | | | Difficult -3 -2 -1 0 +1 +2 +3 easier | | | | | | |
| 47. | Lack of information (training/education) about how to implement would make it________ for me to implement a sustainable solid waste management practice in the households, for the next 12 months . | | | Difficult -3 -2 -1 0 +1 +2 +3 easier | | | | | | |
| 48. | Lack of equipment availability would make it------------- for me to to implement a sustainable solid waste management practice in the households, for the next 12 months . | | | Difficult -3 -2 -1 0 +1 +2 +3 easier | | | | | | |
| 49. | To me, the workload would make it_______to implement a sustainable solid waste management practice in the households, for the next 12 months . | | | Difficult -3 -2 -1 0 +1 +2 +3 easier | | | | | | |
| **J.** | **Behavioral Intention** | | | | | | | | | |
| 50. | I intend to implement a sustainable solid waste management practice in the households, for the next 12 months . | | | Unlikely -3 -2 -1 0 +1 +2 +3 Likely | | | | | | |
| 51. | I am determined to implement a sustainable solid waste management practice in the households, for the next 12 months . | | | Disagree -3 -2 -1 0 +1 +2 +3 Agree | | | | | | |
| 52. | I have decided to implement a sustainable solid waste management practice in the households, for the next 12 months . | | | Disagree -3 -2 -1 0 +1 +2 +3 Agree | | | | | | |

Facilitator name______________Signature________________ Date ___/__/_____

Checked and approved by Supervisor name__________Signature_____ Date ___/__/____

Cross checked and approved by investigator name Semu Debebe Fikadu . Sign. _Date ___/__/___
