# Supplementary material for: Intention to comply with solid waste management practices among households in Butajira town, Southern Ethiopia using the theory of planned behavior
Source: PLoS One. 2022 Jul 8;17(7):e0268674. doi: 10.1371/journal.pone.0268674 (PMC9269971; doi:10.1371/journal.pone.0268674)
Supplement: Attachment — Submitted filename: PLOS nov 2021.pdf [file pone.0268674.s003.pdf]

Review Results

Manuscript Title: Intention to Comply with Solid Waste Management Practices among households in Butajira Town, Ethiopia using the Theory of Planned Behavior

No.	Section	Comments
1.	Title	The title needs to be further improved. Too long.
2.	Abstract	The abstract is good and has most of the important points that need to be highlighted. However, the main findings are not well explained. The contribution of this study to theory/body of knowledge not clear.
3.	Introduction	The introduction highlights the issues of waste management and gap of the study. However, more details are needed on statistics of waste in Ethiopia.<br>I would suggest the author to use the new TPB instead. In the new TPB, perceived behavior control is the moderator and not predictor for intention. Revisit this issue.<br><br>The used of the old TPB is no longer acceptable. Provide strong reason why the old theory of TPB was used in this study?
4.	Methodology	Sample size determination need to be elaborated clearly.<br>More detail elaboration is needed on the data collection procedure, measurement items need to be clearly explained. Perhaps the author needs to state clearly the measurements item. For example, need to state whether the items have been adopted or adapted, and from where, indicate source and year for all the items.<br><br>Revisit the results section. Results section has not been presented clearly. Results of the multiple regression were not clear. Revisit the presentation of the multiple regression results.<br><br>Why only focus on 1 area? The scope of this study is too narrow.
5.	Conclusion<br><br>Theoretical and Managerial Implications	The conclusion section was clearly explained.<br><br>This section discusses about managerial implications but the discussion on the theoretical implications of this study is lacking.<br><br>The limitations and suggestions for future research were highlighted briefly.
6.	References	The number of references were not satisfactory, too few citations and references.
7.	Overall	The article focuses on an important issue but it requires a major revision. Moreover, the contribution of this study to theory is not clear. How this study adds or extends TPB? What is new in this study? Clear justification is needed.<br>Moreover, this study only focused on 1 area, this limits the contribution of this study as well.
