# Supplementary material for: Intention to comply with solid waste management practices among households in Butajira town, Southern Ethiopia using the theory of planned behavior
Source: PLoS One. 2022 Jul 8;17(7):e0268674. doi: 10.1371/journal.pone.0268674 (PMC9269971; doi:10.1371/journal.pone.0268674)
Supplement: Attachment — Submitted filename: Response to reviewers.docx [file pone.0268674.s004.docx]

Date 27 December, 2020

**Dear Academic Editor and Reviewers,**

Thank you so much for your valuable comments and interest in the publication of the manuscript. Your comments have improved the quality of our manuscript and almost all the comments are incorporated into the current manuscript. The "**Revised Manuscript with Track Changes**" will show how much we improved our manuscript as per your comment.

We are pleased to have an opportunity to revise our manuscript, entitled "Intention to Comply with Solid Waste Management Practices among Households in Butajira Town, Southern Ethiopia Using the Theory of Planned Behavior." [PONE-D-21-28599]. In the revised manuscript, we have carefully considered the editors' and reviewers’ comments and suggestions. As instructed, we have attempted to succinctly explain the changes made in reaction to all comments. We reply to each comment in point-by-point fashion. We have color-coded the revised manuscript as text. The responses to the concerns raised by the editor and reviewer are below and are italic coded as follows. The editor’s and reviewers’ comments were very helpful overall, and we are appreciative of such constructive feedback on our original submission. After addressing the issues raised, we feel the quality of the paper has greatly improved.

Note: Datasets used and analyzed during the current study are available from the corresponding author on reasonable request.

**Responses to Editor**

**Q##:** Please ensure that your manuscript meets PLOS ONE's style requirements,

***Response: Thank you for the comment.*** ***We corrected the manuscript in order to meet PLOS ONE's style requirements.***

**Q##:** Please include additional information regarding the survey or questionnaire

***Response: We thank you so much for your comments and refining our manuscript for publication. Both English and Amharic version questionnaire is added.***

**Q##:** Please provide additional details regarding participant consent.

***Response: Consent form is there in the manuscript and added as supplementary materials too.***

**Q##:** Please clarify the sources of funding (financial or material support) for your study.

***Response: Elaborated in the text of manuscript where appropriate.***

**Responses to Reviewer #1**

Thank you for your valuable comments and interest on our manuscript for publication. Your comments were incorporated into the revised manuscript and clarified one by one.

**Q##:** What is the main contribution of this study to the body of knowledge? contextual contribution is not acceptable.

***Response: Thank you for the valuable comment. The outcome of this study will provide to the Federal Ministry of Health and other health organizations with a comprehensive understanding and strategies on the households’ behavioural intentions to be engaged in the solid waste management practice. The guiding principles extracted from the findings will be useful for researchers, policy-makers, practitioners and other public health experts in Solid Waste Management system.***

**Responses to reviewer #2:**

Thank you very much for your comments and refining our manuscript for publication. Your comments were incorporated into the revised manuscript and clarified one by one.

**Q##:** Methodology could be a bit unclear. Introduction: would be good to define what is included under the solid waste management activities/practices to be complied with? e.g. waste segregation, recycling or home composting? Is there any specific regulation/policy on waste management practices by the country/city?

***Response: Corrected. Thank you for the valuable comment.***

**Q##:** Materials and Methods: measurement definitions: what are the 3 items and 4 items etc referring too? Suggest can elaborate briefly or in brackets for further clarity. This section is quite lengthy. Kindly consider breaking into shorter paragraphs and with a flowchart diagram showing the progression of scoring, if applicable.

***Response****:* ***We thank you so much for the valuable comment. Since the constructs of theory of planned behaviour are scale variables, it is mandatory to use some items or parameters that can measure the constructs of theory of planned behaviour. So, these items refer some parameters that were used to measure the constructs.***

**Q##:** Materials and Methods: variables definitions: suggest that the variables can be further elaborated and defined. e.g. what does it mean or is included under indirect attitude?

***Response****:* ***Thanks for the comment. Attitude measurement is an attempt to convert observations of a person's behaviour toward a referent into an index representing the presence, strength, and direction of the attitude presumed to underlie the behaviour. Direct methods to measure attitude are those in which the respondents are either informed that their attitudes are being measured or are made aware of it by the nature of the attitude measurement technique. It is assumed that the respondent and the researcher view the experimental task similarly and have attached the same meaning and significance to the response that is requested. In contrast to the direct methods, indirect methods to measure attitude yield responses that are not taken literally. Rather, the respondent's performance on an overt and seemingly straightforward objective task is thought to unconsciously reveal latent psychosocial constructs that are interpreted as attitude.***

**Q##:** Materials and Methods: data entry and analysis: why does a p-value of less than 0.25 was chosen? As co-linearity among parameters could pose some issues for regression analysis, is there any approaches/strategies in addressing this?

***Response:*** ***We incorporated your valuable comment in the current manuscript.* *Variance Inflation Factors (VIFs) measures the extent to which multi-collinearity has increased the variance of an estimated coefficient. It looks at the extent to which an explanatory variable can be explained by all the other explanatory variables in the equation. The VIF for each independent variable can be obtained by regressing it against all others in the set being analysed, and then calculating (1/[1 − R^2^]). A VIF of 1.8 tells us that the variance of that predictor variable (i.e. its standard error) is 80% greater than would be the case with no collinearity effect: VIFs of 2.5 or greater are generally considered indicative of considerable collinearity suggesting that there will be difficulty in separating out the independent contribution of variables with such large VIFs—although some authors. Some strategies that can solve collinearity problems are: dropping a Redundant Variable, Transforming the Multicollinear Variables and Increasing the Sample Sizes. We used a p-value of less than 0.25 in bivariate regression analysis because most of the references used this cut off point to identify variables that are eligible for multiple linear regression analysis.***

**Q##:** Results: pg 10: the r= 0.29 or 0.38 seems not to be a high correlation strength... Is there some mechanisms/literature to discuss further on the relationship?

***Response: Well, thanks! To improve the correlation coefficient, one of the mechanisms is increasing the difference between the variables. This is done by identifying the independent variable observation, which is same or close to dependent observation value, and replacing it with the value which would increase the difference between the variables.***

**Q##:** Results: pg 16: some of the observations were reported by only 1 participant. Would be good also if the discussion can be added on with more representative observations.

***Response: Corrected with great thanks.***

**Q##:** Table 3 pg 18: where does 0.71 derived from as it was not observed in table 3? Why the variable with beta 0.09 does was chosen as it was stated as not significant in the text? Would be good to discuss/clarify the selection of variables for the final analysis.

***Response:*** ***Corrected with great thanks***

**Responses to reviewer #3**

Authors would like to thank you for your comments and refining our manuscript for publication. Your comments were incorporated into the revised manuscript and answered one by one.

**Q##:** It is also important to illustrate the current status of municipal solid waste management in Ethiopia through statistical data, which can help readers understand why Ethiopian cities can be used as research objects. In addition, it is recommended to elaborate on the reasons for choosing Butajira Town as the specific research area in the Study area and period section.

**Response: *Corrected***

**Q##:** the selection process of the research objects of the elicitation study should be explained.

***Response: Corrected***

**Q##:** In the section of Ethical considerations and informed consent, the authors obtained the permission from the Butajira health office on June 7, 2012, but the research period is from June 1 to 30, 2020. Please confirm that this time of permission is correct. Too long a time interval will cause confusion, and it is recommended to delete the content of permission.

***Response: Corrected, June 7, 2012 was in Ethiopian calendar, changed to G.C. (June 19, 2020)***

**Q##:** also the meaning of intention variables must be clearly explained

***Response: We thank you for the comment and elaborated the meaning of intention variables.***

**Q##:** In the section of Measurement, variables and operation definitions, the authors use three items on the Semantic Difference Scale to measure the intention of the solid waste management practice, but fails to explain the content of the items in the scale.

**Response: *Corrected***

**Q##:** Although the authors divided attitude, subjective norm, and perceived behaviour control into two categories of direct and indirect, they did not present the content of the relevant items in the scale on the manuscript. In order to understand the meaning of the variables easily, it is recommended to select appropriate items in the scale to explain the variable.

***Response: Corrected (we narrated this issue under measurement of variables in the manuscript). Thanks for the comment.***

**Q##:** It is necessary to explain why there is no correlation between the direct and indirect measures of subjective norms.

***Response: Corrected and explained in the text.***

**Q##:** The authors should state their research hypothesis in the appropriate place of the manuscript.

***Response: Stated with thanks!***

***Thank you very much to editor and reviewers for your precious comments!***
