# Supplementary material for: Intention to comply with solid waste management practices among households in Butajira town, Southern Ethiopia using the theory of planned behavior
Source: PLoS One. 2022 Jul 8;17(7):e0268674. doi: 10.1371/journal.pone.0268674 (PMC9269971; doi:10.1371/journal.pone.0268674)
Supplement: Attachment — Submitted filename: Response to reviewers.docx [file pone.0268674.s005.docx]

**Date:** 08 April, 2022

**Dear Academic Editor and Reviewers,**

We thank you so much for your valuable comments and interest in the publication. **We revised the comments of reviewer 2.** The "**Revised Manuscript with Track Changes**" will show how much we improved our manuscript as per your comment.

We are also pleased to have an opportunity to revise our manuscript, entitled "**Intention to Comply with Solid Waste Management Practices among Households in Butajira Town, Southern Ethiopia Using the Theory of Planned Behaviour" [PONE-D-21-28599]**. The comments are very important to improve the quality of paper. We used track-changes to reply each concern as per your suggestion, and put below one by one. We believe the paper's quality has considerably improved after we addressed all of the issues identified.

**Responses to Editor**

- We considered the points you suggested to meet PLOS ONE's style requirements while you are preparing the manuscript.

**N.B:** Datasets used and analyzed during the current study are available from the corresponding author on reasonable request.

**Responses to Reviewer #2**

Thank you for your valuable comments and interest in our manuscript for publication. Your comments were incorporated into the revised manuscript and clarified one by one.

***Q (1).*** Introduction: would suggest elaborating briefly on the studies applying TNB on waste management, e.g. the approaches, findings or limitations on the respective studies, instead of having lumped references, so that the research gap and the objective of the current work can be better highlighted.

***Answer: Your comment is incorporated in the revised document. Thank you for the valuable comment.***

***Q(2).*** Research hypothesis: abbreviation PCB not defined.

***Answer: We defined the abbreviation for the first time as Perceived Behavioural Control (PBC). Thank you for the comment.***

***Q(3).*** Methodology- Study design & Measurement, variables and operation definitions: for the 'solid waste management practice' and 'sustainable solid waste management practice' mentioned, are there any specific activities being referring to? e.g. waste segregation, home composting, collection etc.

***Answer:*** **Thank you for the concern and comments. Our research aim is to assess the intention of the households on the variables you listed under solid waste management, not to measure each variable in a practical manner. However, we put the operational definitions in the current document.**

**Q(4).** Methodology- Data entry, processing and analysis: how is unstandardized β coefficient calculated and why is it chosen over standardized β coefficient?

**Answer:** ***Thank you for the comment. It is really interesting***. ***β is unstandardized coefficients which means original units besides the slope and tell if the independent variable is a significant predictor of the dependent variable. Beta is a standardised coefficient between -1 to +1 in range and show the strength of the prediction. Unlike standardized coefficients, which are normalized unit-less coefficients, an unstandardized coefficient has units and a 'real life' scale. An unstandardized coefficient represents the amount of change in a dependent variable intention to solid waste management due to a change of 1 unit of independent variable (socio-demographic variables and others). In other words; unstandardized coefficients are obtained after running a regression model on variables measured in their original scales. Standardized coefficients are obtained after running a regression model on standardized variables (i.e. rescaled variables that have a mean of 0 and a standard deviation of 1).***

***Q(5*).** Under Ethical considerations and informed consent, the reference number for the support letter appeared to have three empty boxes. Is it a typo? Kindly check.

***Answer: We corrected in the revised manuscript. Thank you!***

**Q(6).** R2 not subscripted. Kindly check.

***Answer: Thanks for the comment. We corrected in the current document.***

***Q(7).*** Suggest the conclusion paragraph can be under a new section Conclusion.

***Answer: Thank you so much for the comment. We put the conclusion paragraph in a new section.***

***Thank you very much to editor and reviewers for your valuable comments!***
